# Contrafreeloading Indicating the Behavioural Need to Forage in Healthy and Feather Damaging Grey Parrots

**DOI:** 10.3390/ani13162635

**Published:** 2023-08-15

**Authors:** Yvonne R. A. van Zeeland, Nico J. Schoemaker, Johannes T. Lumeij

**Affiliations:** Division of Zoological Medicine, Department of Clinical Sciences, Faculty of Veterinary Medicine, Utrecht University, 3584 CM Utrecht, The Netherlands

**Keywords:** *Psittacus erithacus*, psittacine, foraging, enrichment, feather damaging behaviour, feather plucking, feather picking

## Abstract

**Simple Summary:**

When given the choice, many animals will opt to put in effort to obtain their food even when the same food is readily available in a food bowl nearby. This behaviour, termed contrafreeloading, illustrates the importance of providing so-called foraging opportunities, which allow animals to search and work for food, much like their wild counterparts. However, in animals with abnormal behaviours, e.g., parrots with feather damaging behaviour (FDB), this motivation to work for food may no longer be present. This study therefore aimed to determine whether healthy and feather damaging parrots differ in their motivation to contrafreeload by offering them the choice between “free food” from a food bowl or “earned food” that needed to be extracted from a foraging device. Feather damaging parrots were found to spend less time and consume lower amounts of food from the foraging device, demonstrating they were indeed less inclined to work for food than healthy birds. Nevertheless, all birds used the foraging device, and of those with FDB that used the device the most, plumage also improved the most. These findings emphasize that foraging opportunities should be provided to captive parrots to satisfy their motivation to work for food and forage.

**Abstract:**

Contrafreeloading (CFL) is a concept that describes the preference of an animal to work for food even when identical food is freely available, and reflects an intrinsic motivation to engage in foraging-related activities. However, altered brain neurochemistry, which can be induced by chronic exposure to a suboptimal living environment, may affect this intrinsic motivation in animals with abnormal repetitive behaviours (ARBs), including parrots with feather damaging behaviour. To determine whether this was the case, we evaluated CFL activity in healthy (*n* = 11) and feather damaging (*n* = 10) Grey parrots (*Psittacus erithacus*) by offering them a free choice to obtain identical food from a food bowl or from a foraging device. Differences in CFL activity were observed, with feather damaging Grey parrots displaying less CFL (as indicated by shorter foraging times and lower amounts of food consumed from the foraging devices) compared to healthy conspecifics, indicating altered ‘motivation’ and time allocation, for which the underlying mechanism needs to be clarified further. Nevertheless, despite the variable level, all birds displayed CFL, which, together with a seemingly positive correlation between CFL activity and the plumage condition of the birds, suggests that parrots are intrinsically motivated to forage and highlights the importance of providing foraging opportunities to captive parrots.

## 1. Introduction

Contrafreeloading (CFL) is a behavioural phenomenon that reflects an animal’s preference to work for food (“earned food”) even though identical food is freely available from another, nearby source (“free food”) [1]. After Jensen [2] first described occurrence of CFL in rats (*Rattus norvegicus*) in the 1960s, the phenomenon has been widely demonstrated in both wild and domesticated animal species, including mice (*Mus musculus*) [3], gerbils (*Meriones unguiculatus*) [4], mink (*Mustela vison*) [5], pigs (*Sus scrofa*) [6,7], cattle (*Bos taurus taurus*) [8], goats (*Capra aegagrus hircus*) [9], Northern giraffes (*Giraffa camelopardalis*) [10], Grizzly bears (*Ursus arctos horribilis*) [11], Maned wolves (*Chrysocyon brachyurus*) [12], Chimpanzees (*Pan troglodytes*) [13], Japanese and Rhesus macaques(*Macaca fuscata*, *Macaca mulatta*) [14,15], Siamese fighting fish (*Betta splendens*) [16], American crows (*Corvus brachyrhynchos*) [17], starlings (*Sturnus vulgaris*) [18], Rock pigeons (*Columba livia*) [19], chickens (*Gallus gallus*, *Gallus domesticus*) [20,21], and various types of parrots, including Orange-winged Amazon parrots (*Amazona amazonica*) [22], Grey parrots (*Psittacus erithacus*) [23,24] and Kea (*Nestor notabilis*) [24]. Contrafreeloading is usually explicitly studied by providing animals a choice between free food in a food bowl and performing an operant (e.g., lever press) or natural foraging task (e.g., scatter feed, manipulate a foraging device) to obtain the same type of food. However, CFL has also been observed as an ‘incidental finding’ during other studies on environmental enrichment [22,25].

Some authors [26,27] have proposed that CFL only occurs if the proportion of “earned food” exceeds 50% of the total amount of food consumed during a trial or session. However, there are no obvious reasons to assume 50% as the cut-off point for CFL as animals were also found to choose to work for resources that are not (completely) consumed [11,15,19,28,29,30]. Moreover, choosing to work for food contradicts optimal foraging theory as this theory states that animals will always strive to maximize reward or energy gained while minimizing effort or cost [31], thereby predicting an absolute preference for “free food”. Hence, CFL is more commonly defined to occur whenever the animal chooses to work for a resource in the presence of free availability of the same item, regardless of the amount [1,11,12,18].

Over the years, many authors have speculated why animals contrafreeload. Explanations for this behaviour include stimulation seeking [1], opportunity to play [23,24], alleviation from boredom [11], reducing uncertainty in an unfamiliar (captive) environment [1,11], innate reinforcing properties of the behaviour, particularly where it concerns species-typical behaviours [1], and gathering information about the environment and potential alternative food sites as part of the so-called ‘information primacy hypothesis’ [1,32,33]. The information primacy hypothesis suggests that foraging decisions involve a dynamic process in which the additional energy spent on working for food is weighed against the satiation of immediate needs in the short term and the possibilities for more efficient food intake due to decreased environmental uncertainty in the long term [1,32,33]. As such, food deprivation or an increase in the effort required to obtain the food can negatively affect CFL [1,28,34,35,36,37]. 

Other factors that have been suggested to influence CFL include age [11,27], sex [38,39], body condition [10], production level (in chickens) [20,40,41], prior training [1,2,42,43], duration or repetition of test trials [1,44,45], and adverse living conditions, including social isolation [27], increased food competition [39], and sensory deprivation, both during early life and adulthood [1,46,47,48,49]. Previously, Garner [50] also suggested that stereotypic and other abnormal repetitive behaviours (ARBs) that are inappropriate, repetitive and unvarying in either goal or motor pattern [51], are likely to affect choice or decision making tasks, including CFL. In humans [52,53,54] and animals [55,56,57,58,59], ARBs have indeed been correlated with so-called ‘perseveration’ (i.e., continuation or recurrence of a response or activity without the appropriate stimulus), as evidenced by the responses observed during neuropsychological tests (e.g., gambling task, extinction task). However, none of the research so far seems to have studied the actual effects of behavioural pathology on CFL.

Feather damaging behaviour (FDB) is one of the behavioural disorders that can be classified as an abnormal repetitive behaviour. Feather damaging behaviour, which occurs in approximately 10–15% of captive parrots [60,61,62,63], involves (repetitive) plucking or damaging of the feathers on body areas that are accessible to the bird’s beak (e.g., chest, neck, and ventral wing surface) [64,65,66]. Similar to various other behavioural disorders, FDB is considered as a multifactorial disorder in which various socio-environmental (e.g., social isolation, lack of foraging opportunities), neurobiological (e.g., serotonin, dopamine, corticosterone, sex hormones), medical (e.g., atherosclerosis, osteoarthritis, bacterial folliculitis, avian ganglioneuritis), and possibly genetic factors may play a role [67]. 

An important environmental feature involved in the development of FDB includes the lack of foraging opportunities. In fact, various researchers have postulated that FDB should be considered as ‘redirected foraging’ behaviour [68,69,70]. As a result of a lack or limitation of foraging opportunities in the captive environment, the time spent on foraging is drastically decreased. Whereas wild parrots spend between 4 and 6 h on searching, selecting, and manipulating food [71,72,73,74,75], captive birds usually consume their food in less than an hour [22,76,77,78]. The lack of appropriate target stimuli to engage in this type of species-specific behaviour may subsequently result in the development of FDB [67,79,80,81]. Indeed, a correlation has been found between FDB and the provision of foraging opportunities. Both Meehan et al. [69] and Lumeij and Hommers [70] demonstrated that feather damaging behaviour diminished when birds were presented with foraging devices. In addition, Meehan et al. [69] showed a preventive effect of offering foraging opportunities on the severity of feather damage, as the birds that were presented with foraging opportunities maintained a better feather score than birds that were denied the opportunity to forage, until deprived of such opportunities themselves [69]. 

The observed correlation between foraging opportunities and FDB in the aforementioned studies could be explained from two perspectives: (1) the change in FDB is extrinsically motivated, i.e., birds merely used the foraging devices as these were the only option to obtain food, thereby preventing them from spending time on other activities such as FDB; or (2) the change in FDB results from the fulfilment of an intrinsically driven ‘behavioural need’ (i.e., a strong internal motivation to display species-typical behaviours such as foraging [82,83]), which prompted the use of foraging devices. Because CFL reflects an internal motivation to work for food or forage, studying CFL in (feather damaging) parrots could provide clues to help answer the question of whether foraging is a ‘behavioural need’ for parrots. After all, if the decrease in FDB is the result of the fulfilment of a ‘behavioural need’ that previously was not met, parrots with FDB, when given a (free) choice, are expected to spend equal amounts of time on foraging and show similar improvement in feather condition to those that were ‘forced’ to forage by Lumeij and Hommers (who offered foraging devices as the parrots’ sole food source). 

Initially, lack of foraging opportunities or other features related to the environment may lead to the onset of ARBs in an attempt of the animal to cope with and adapt to the suboptimal conditions of its captive environment with functionally intact behavioural mechanisms [84]. However, long-term exposure to such conditions, as well as averse early life events may induce abnormal psychology, brain development, or neurochemistry [85,86,87,88]. As a result, the behaviour may become ritualized and malfunctional, occurring in absence of obvious ‘triggers’ [84]. In these particular cases, brain dysfunction leads to ‘perseveration of behaviour’, thereby affecting processes related to switching or suppression of behaviour, including CFL. As such, individuals with ARBs are expected to display less CFL than healthy conspecifics. 

Although previous reports have provided evidence that parrots display contrafreeloading [22,23,24,25,89], it is currently unknown whether feather damaging individuals also contrafreeload, and to what extent. We therefore performed a study on CFL in both healthy and feather damaging parrots. As a model, we selected the Grey parrot as this is one of the species most commonly affected by FDB [62,63,90,91], with a reported prevalence of almost 40% [92]. Grey parrots, also known as African Grey or Congo Grey parrots, are medium-sized, Old World parrots from the family Psittacidae. They are a highly social, intelligent, monogamous, mostly frugivorous species that lives in large groups of up to ten thousand birds in densely wooded rainforest and wooded savanna in equatorial Africa [93,94,95,96,97,98]. Being a non-domesticated species, captive Grey parrots have retained many characteristics of their wild conspecifics [99,100], which places high demands on their captive living environment and likely renders them susceptible to developing FDB in captivity if these demands are not met. With foraging comprising an important part of the Grey parrots’ daily activities [101,102], we hypothesized that (healthy) Grey parrots would display relative high levels of CFL. In addition, based on the link between ARB and perseveration of behaviour, we hypothesized that feather damaging individuals in which the behaviour is chronic and malfunctional would contrafreeload to a lesser degree than healthy birds. Last, we hypothesized that in feather damaging Grey parrots, the improvement in feather condition would be positively correlated with the amount of time spent on CFL for foraging to be a true ‘behavioural need’.

## 2. Materials and Methods

### 2.1. Ethical Approval

This study was approved by the Institutional Animal Care and Use Committee of Utrecht University (DEC 2009.I.09.073). The sanctuary that owned the birds that participated in the study was informed of the design and set-up and consented to the procedures prior to initiating the study.

### 2.2. Animals

Twenty-one unrelated Grey parrots of both sexes, with a median age of 11 years (range 1–27 years) and a mean (±SD) body weight of 487 ± 60 g (range 374–612 g), were included in this study. Ten of these parrots displayed feather damaging behaviours and eleven did not. Originally, the study involved 11 feather damaging birds, but one of the birds died without prior symptoms before inclusion in the study. Post-mortem examination of this bird revealed hypertrophic cardiomyopathy and atherosclerosis. 

All parrots were previously privately-owned birds that were relinquished to a parrot sanctuary (Nederlands Opvangcentrum voor Papegaaien (NOP), Veldhoven, The Netherlands). Detailed histories regarding the birds’ rearing conditions and living environment prior to their arrival in the sanctuary were not obtained as this information was considered confidential and therefore remained undisclosed. In the sanctuary, the birds were permanently housed together for at least a year in a large outdoor aviary (L × W × H = 8 m × 4 m × 4 m) that was equipped with wooden logs, trees, bushes, branches, several nesting boxes, food and water bowls, and sand as substrate. Prior to the study, foraging opportunities (other than, e.g., scatter feeding and using multiple food stations) were not routinely provided to the birds.

In the group of parrots with feather damaging behaviour, all parrots showed chronic FDB (present for at least 1 year) according to the typical pattern of self-inflicted damage to the feathers, i.e., presence of well-formed feathers on the head and presence of (a varying degree of) feather loss or damage in body areas accessible to the parrots’ beak, and apparently unresponsive to environmental changes. DNA analysis of the birds revealed that this group consisted of 5 male and 5 female parrots, with ages ranging from 2 to 28 years (mean 15 years; including one bird of unknown age). The group of parrots without feather damaging behaviour included 7 males and 4 females, with ages between 1 and 26 years (mean 11 years; including two birds of unknown age). No significant weight differences were present between the two groups. 

Prior to the study, all birds were physically examined and found to be clinically healthy, with no evidence of underlying medical problems for feather damaging behaviour. Throughout the study, the parrots’ health status, plumage condition, activity pattern, and behaviour (including presence of abnormal behaviours such as locomotor, whole-body, or oral stereotypies) were monitored daily. Body weights were recorded weekly. 

### 2.3. Housing and Nutrition

**Figure 1:**
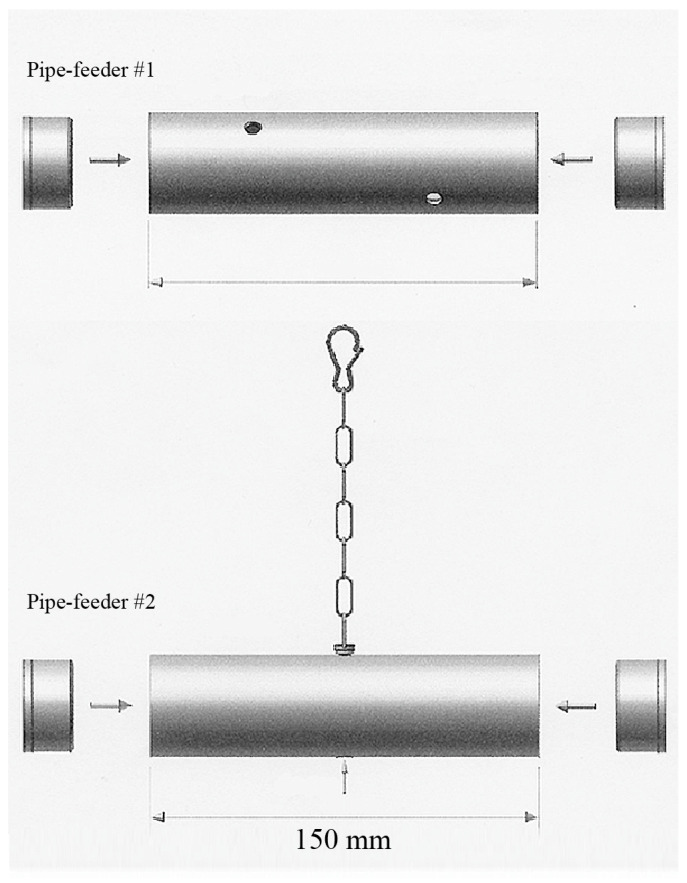
Two pipe feeders (size 150 mm × 40 mm) were used to provide foraging opportunities. Pipe feeder #1 was placed on the bottom of the cage. Parrots had to push and roll this foraging device in order to obtain the pellets via one of the four holes (diameter 7 mm). Pipe feeder #2 was suspended from the ceiling. Parrots had to manipulate the pellets out of the hole in the centre of the bottom, or push against or swing the pipe feeder in order to release the pellets from the device.

**Figure 2:**
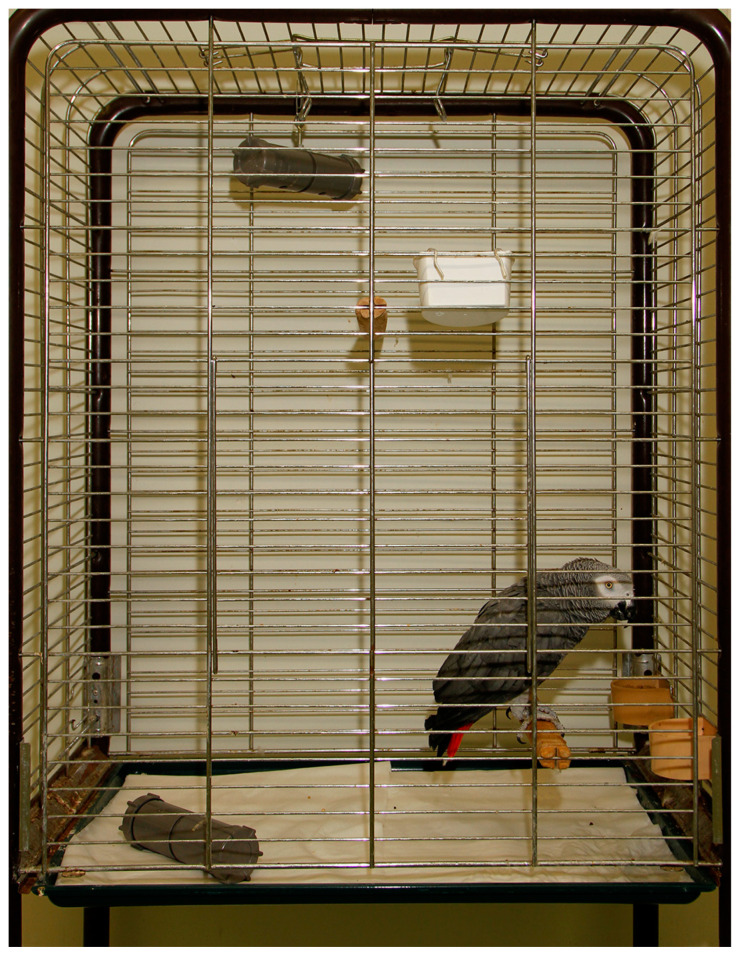
Experimental set-up of the cage in which the parrots were housed.

For the purpose and duration of this study, parrots needed to be housed individually to enable accurate data collection regarding food consumption and foraging times for each individual. Individual housing took place in ‘standard-sized’ parrot cages meeting the minimum housing requirements for Grey parrots (L × W × H = 90 cm × 60 cm × 120 cm) [103]. Each enclosure was furnished with two soft wooden perches, one water bowl, and two food bowls. In addition, two foraging toys (tube-shaped, opaque PVC pipe feeders, Figure 1) were present inside the cage. One food bowl and pipe feeder were located at ground level, whereas the other food bowl and pipe feeder were located at the top of the cage (Figure 2). The soft wooden perches and (news)paper distributed on the floor served as ‘enrichment’ that the parrots could chew and gnaw on. Other than these items, no toys or other enrichment items were provided as birds were not accustomed to these in their regular enclosure, and because any items hanging from the top of the cage or fixed to the walls would have obscured the view of the parrots during the observations.

All parrots were housed indoors in the same room, where they were exposed to 11 h of artificial light per day (from 6.30 a.m. to 5.30 p.m.). Temperature in the room was between 18 and 25 °C and humidity levels varied between 45% and 60%. Visual barriers were installed between the cages to prevent social learning of foraging preferences and tactics. Parrots were able to communicate vocally with each other. In addition, a radio was turned on and off simultaneously with the lights to provide some additional sensory enrichment [104,105]. 

One month prior to the start of the study, parrots were converted from a mixture of pellets (Nutribird P15 Original, Versele-Laga, Deinze, Belgium) and seeds to a full pelleted diet (Scenic Paradise Mix^®^, Scenic Bird Food, Plymouth, MN, USA). Conversion to this specific type of pellet (i.e., round shape with a diameter of 5 mm instead of an elongated flat shape of 13 mm × 8 mm × 5 mm) was necessary in order to ensure that all food particles were completely symmetric and similar in size and shape to facilitate consistent passing of food through the holes in the pipe feeders without clogging the toys. The food and drinking water were refreshed daily around 9 a.m. by a group of three caretakers. These caretakers also cleaned the cages daily before refreshing the food and ensured that they spent a maximum of two hours per day in the rooms where the parrots resided. For the duration of the study, only this limited group of three caretakers was allowed to access the room where the parrots stayed. 

### 2.4. Acclimatization Period

Parrots were allowed to acclimatize to the novel environment simultaneously with the food conversion. In addition, a stepwise protocol was used to teach the parrots how to use and obtain food from the different pipe feeders. After the birds had learnt how to use these foraging devices, which took approximately one week, food (60 g, similar to the study of Lumeij and Hommers [70]) was distributed at random to the parrots in only one of the four items for the remainder of the acclimatization period. For each parrot, the item in which the food was offered changed daily and was determined at random, to ensure the parrots would be able to obtain their daily food consumption from each of the items separately, without inducing a learning or habituation effect.

### 2.5. Contrafreeloading Trial

Following the acclimatization period, the 4-week contrafreeloading trial began, in which four foraging options were presented simultaneously to the birds using a standardized set-up: (1) a free food bowl located in the lower corner of the cage, near a perch; (2) a free food bowl located in the upper, opposite side of the cage, near a perch; (3) a pipe feeder located at the bottom of the cage; and (4) a pipe feeder suspended from the ceiling of the cage. 

Each day, each of the food sources was filled with 60 g of pellets, an amount that, based on observations during the acclimatization period, was determined as sufficient to enable the bird to obtain their daily food intake from one source as leftovers were present in all enclosures during the entire acclimatization period following provision of 60 g of pelleted diet. Birds were subsequently allowed to choose freely from which of the food sources they would want to obtain their food throughout the day. Between 8.30 and 9.30 a.m. the next morning, the remainder of food was removed from each food source and weighed. These values were subsequently used to calculate how much the birds actually consumed from each food source. As the food sources where strategically placed in the cage, it was possible to determine the origin of spilt pellets, when present. The weight of these pellets was subsequently used to correct total food consumption and consumption from the specific sources.

### 2.6. Video Recording and Analysis of the Behaviour

To avoid influence from an observer on the behaviour of the parrots, behaviours were captured on video with the use of surveillance cameras. Two parrots in adjacent cages could be filmed simultaneously. Each day, at feeding time, cameras were repositioned to other enclosures to ensure that each parrot was filmed at least twice per week, resulting in a total of eight recordings. 

As Grey parrots roost at night [95,96], and we rarely found them active and never observed foraging at night in previous studies (unpublished), filming only took place during the daytime period. Nevertheless, we made sure to extend recording for 60 min after lights were turned off and started recording 60 min before lights were turned to check whether the birds were inactive and roosting during this time, and we verified for each recording whether the birds’ roosting location at the end of the video on the one day and start of the video the next day was identical, as this supported our assumption that they indeed had remained inactive throughout the night. 

Video recordings were subsequently analysed for time spent on foraging and preening by each of the parrots by a single rater with over 2 years of experience in analysing parrot behaviour. Foraging time included the total time spent on all behaviours related to foraging (i.e., searching for food, manipulation of foraging devices, grabbing food directly from the holes in the pipe feeders, climbing down to pick up pellets from the ground, picking up food from the food bowl, podo-mandibulation (manipulation of pellets with beak and tongue while holding them with the foot), consumption of the food). Preening time included the total amount of time spent on behaviours related to manipulation of the plumage with the beak (i.e., nibbling, stroking, and rubbing of feathers). Behaviours that involved care of the feet, nails, or beak were not included. Within-observer agreement was determined using the same methodology as described by Beekmans et al., 2023 [106], with ICCs > 0.90 for all behaviours, hence representing excellent agreement [107]. 

In addition to analysing total foraging time, foraging times per individual parrot were analysed and calculated separately for each of the sources from which the birds could obtain the food. Some birds would occasionally climb down to search and consume food that had been spilt or dropped on the floor. Since this foraging time could not be allocated to any specific food source, it was calculated separately, unless pellets were picked up directly after dropping them on the floor. In the latter case, the ground foraging time was included in the time spent on foraging from the source from which the food originated.

### 2.7. Analysis of Contrafreeloading

Given the generally accepted definition of CFL as any level of effort invested in obtaining a resource when the same resource is also available in an easy accessible form [1,11,12,18], we defined CFL to occur whenever the birds chose to manipulate the foraging devices instead of using the food bowl in which the same type of food was presented. Using this definition, the investment in CFL by each parrot was expressed in both percentage of total food intake and percentage of total foraging time spent on foraging from the pipe feeders. 

### 2.8. Plumage Scoring

Plumage scoring was used as an indirect measurement for (a change in) feather damaging behaviour. To quantify the plumage condition of the different parrots, a feather scoring system was used, scoring (1) coverts and down feathers of five separate body parts (i.e., front (neck, chest, flank), back, legs, and dorsal and ventral surface of the wings); and (2) flight (primaries, secondaries) and tail feathers (Table 1 and Supplementary Materials). 

Scoring of the plumage condition of the parrots took place at three distinct moments during the study: (1) at the start of the acclimatization period; (2) at the end of the acclimatization period and beginning of the contrafreeloading trial; and (3) at the end of the contrafreeloading trial. As the time in between measurements (i.e., 4 weeks) was too short to include a full moult and subsequent changes in the (damaged) flight or tail feathers, scoring of these feathers was not included in the overall plumage condition score. To ensure bias from recognition (i.e., knowledge about the moment in time at which the plumage condition was scored), photographs were taken of the different body parts of the parrots according to a standardized protocol [108]. At the end of the contrafreeloading trial, all collected photographs (i.e., different time points, parrots, and body parts) were mixed, coded (to ensure a blinded process), and scored by a single rater, who was experienced in evaluating feather scores using this feather scoring system, and in a prior study [108] was found to have an intra-class coefficient (ICC) of 0.94, representing excellent intra-rater agreement [107]. After the scoring of all body parts from all birds and all time points, scores for the various body parts were combined into an overall score for plumage condition for each individual parrot and time point.

### 2.9. Statistical Analysis

All analyses were performed using IBM SPSS software (version 20.0). Unless stated otherwise, data are expressed as mean ± SD and the probability level accepted for statistical significance was *p* < 0.05. For primary parameters of interest with *p*-values that were just above the accepted level of significance (0.05 < *p* < 0.10), post hoc power analyses were performed on the obtained results, using a power of 0.80 and an α of 0.05, to determine the minimum sample size needed to reach a level of statistical significance. 

For the total amount of food consumed, amount of food consumed from the food bowls and pipe feeders, total time spent on foraging, time spent on foraging from food bowls and pipe feeders, and time spent on preening (including duration of preening bouts), initial exploration of the data revealed no obvious effect of test day. Hence, the data obtained over the 4-week period were averaged for each parrot to level out day-to-day fluctuations. Because of the small sample size, we subsequently used the non-parametric Mann–Whitney U test to test for significant differences between the groups of healthy and feather damaging birds. In addition, we used the Wilcoxon signed rank test to determine which food source was preferred (high- versus low-positioned). A Pearson’s r correlation coefficient was used to determine the level of correlation between the average CFL time and amount of food consumed via CFL for each individual parrot.

Differences between the birds with and without FDB in the degree to which they displayed CFL, expressed as % of total food consumption and % of total foraging times, were analysed using a linear mixed-effect model (LMM), with FDB and day included as the fixed and random repeated effect, respectively. In addition, we ran a linear mixed model analysis that also included age and sex, and used the interactions between the variables as the fixed effect to exclude bias from these variables, as these have been found to affect CFL in other animals [11,27,38,39]. Assumptions of the model (linearity of the results, normality of residuals, random intercepts and slopes, independence of the residuals) were checked prior to interpreting the results of the model.

To determine the influence of CFL on change in plumage condition, a linear regression analysis was performed to identify the level of contribution of the total foraging time and time spent on CFL to the change in plumage condition (determined as the difference between feather scores at the beginning and end of the 4-week test period). As no differences were seen in the group of birds without FDB, only birds with FDB were included in this analysis.

## 3. Results

### 3.1. Food Intake

Throughout the test period, food intake fluctuated from day to day for each of the parrots. Intra-individual variations were, however, relatively similar for the different parrots (mean ± SD = 22 ± 4%, *n* = 21). Overall, daily food intake remained stable, with no decreasing or increasing trends identified. On average, birds consumed 68 ± 14 g of pellets per day (*n* = 21). No significant differences were found in total daily food intake between parrots with and without FDB (65 ± 11 versus 69 ± 19 g/day; Mann–Whitney U test: U = 0.043 *n* = 21, *p* = 0.835).

### 3.2. Foraging Times

The parrots’ foraging activities occurred throughout the entire daylight period, with two distinct peaks in activity found during the morning (between 6:30 and 11 a.m.) and late afternoon (between 2 and 5:30 p.m.). On average, birds spent 191 ± 77 min per day (29 ± 12% of the daylight period, *n* = 21) on foraging. Although intra-individual variations in foraging times were quite similar to the variations found in food intake (18 ± 12% versus 22 ± 4%, *n* = 21), inter-individual variation was found to be considerably larger (41% versus 22%, *n* = 21). However, total foraging times of the parrots with FDB (154 ± 61 min/day, *n* = 10) did not differ significantly from those of parrots without FDB (208 ± 79 min/day, *n* = 11; Mann–Whitney U test: U = 2.376 *n* = 21, *p* = 0.123). 

### 3.3. Choice and Preference for Foraging Options

**Figure 3:**
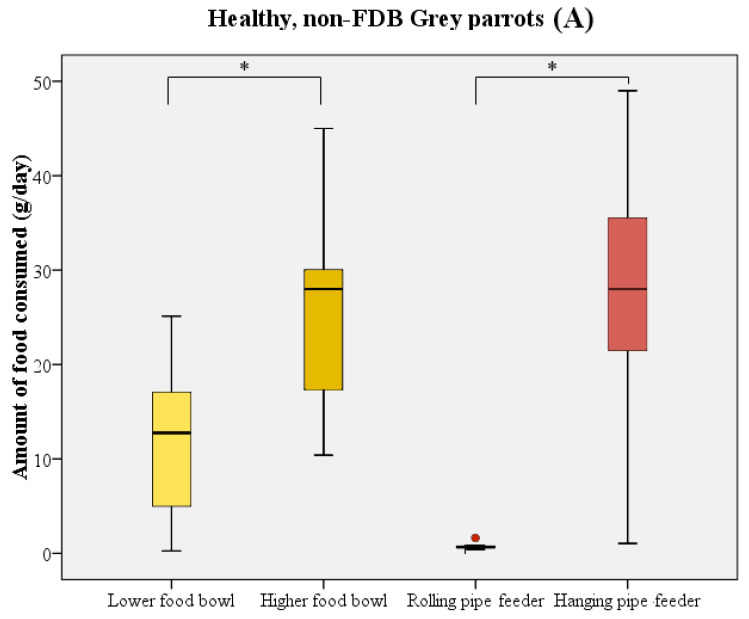

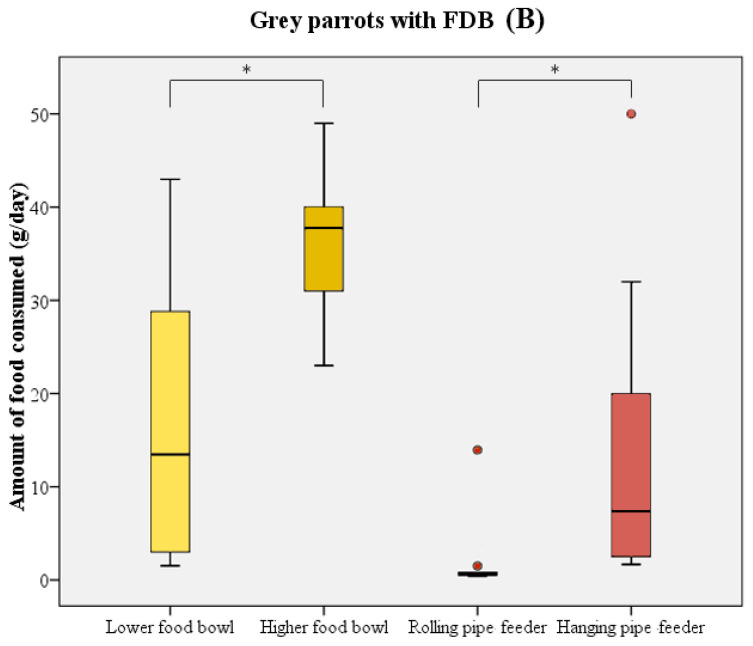
Boxplot representing the food consumption (in g/day) from the food bowls (yellow) and pipe feeders (red) by healthy parrots (*n* = 11, (**A**)) and parrots with FDB (*n* = 10, (**B**)). Boxes, whiskers, and dots represent the interquartile range (IQR); minimum and maximum values (1.5 × IQR); and outliers, respectively. Median values are represented by the horizontal lines within the boxes. Both the healthy birds and feather damaging birds showed a distinct preference for higher-placed food sources. * *p* < 0.001.

The majority of birds directly started to consume food upon receipt of the new food supply. In most cases, parrots initially chose one of the food bowls and consumed a quantity of food from these prior to using the pipe feeders as their food source. During the test period, birds consumed significantly more food from the higher-placed food bowl compared to the lower-placed food bowl (Wilcoxon signed rank test: W = 211 *n* = 21, *p* < 0.001; Figure 3). The higher-placed food bowl was preferred by 14 birds, in comparison to 2 birds preferring the lower-placed food bowl. Five birds did not show a distinct preference for either of the bowls. A similar preference was noted when comparing the two pipe feeders (Wilcoxon signed rank test: W = 223 *n* = 21, *p* < 0.001; Figure 3). Only 1 bird used the rolling pipe feeder compared to 14 out of 21 birds using the hanging pipe feeder. The other six birds, of which five were feather damaging individuals, either consumed less than 5 g/day from the pipe feeders or did not use them at all.

### 3.4. Investment in Contrafreeloading

**Figure 4:**
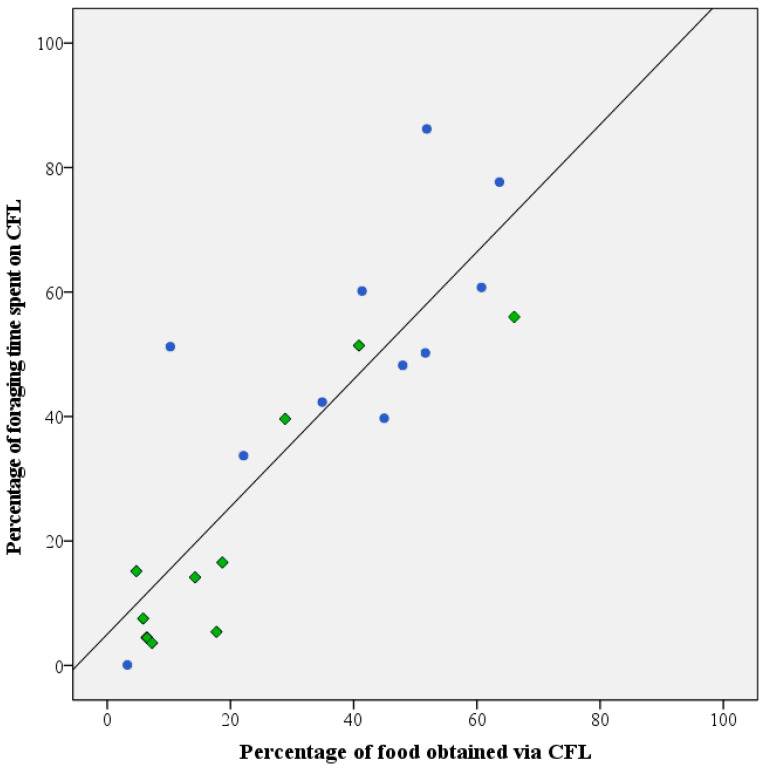
Correlation between CFL as expressed by percentage of daily food intake obtained from the pipe feeders and amount of time spent on foraging from these devices in healthy (●) and feather damaging Grey parrots (◆). An overall strong and significant correlation (Pearson’s r = 0.854 *n* = 21; *p* < 0.01) was detected for the two parameters.

Throughout the test period, daily fluctuations occurred in the two parameters used to assess investment in CFL (i.e., the percentage of foraging time invested in foraging from the pipe feeders and the percentage of food consumed from the pipe feeders. However, upon evaluation of the averaged data for the individual birds, the two parameters were found to be highly correlated (Pearson r correlation: r = 0.854, *n* = 21, *p* < 0.01; Figure 4).

**Figure 5:**
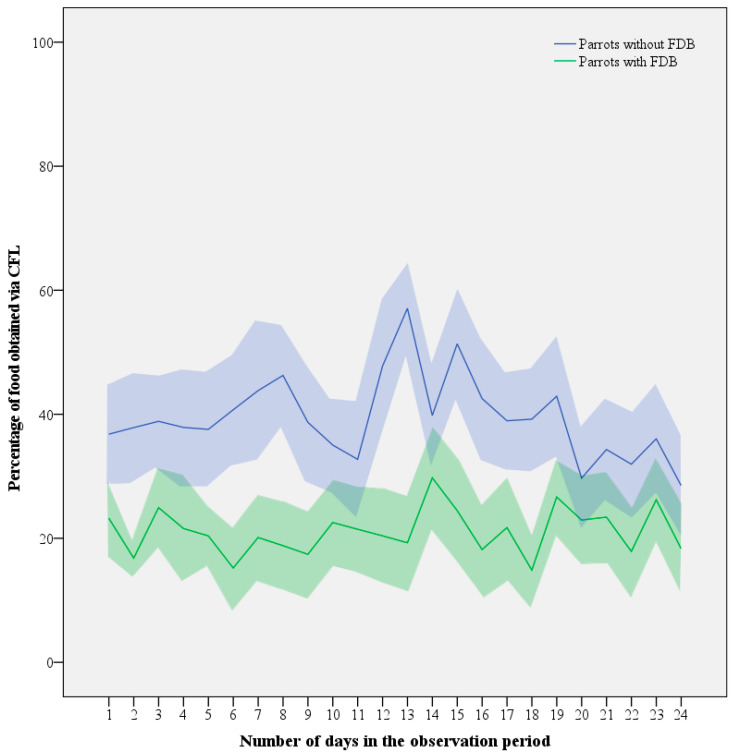
Mean (line) ± SEM (shaded area) investment in CFL expressed as the percentage of food consumed from the foraging devices by Grey parrots with (*n* = 10) and without FDB (*n* = 11) throughout the 4-week contrafreeloading trial.

Despite the daily fluctuations, an LMM revealed no obvious decreasing or increasing trends in time (LMM: F_1,70_ = 0.311, *p* = 0.579; Figure 5). Increasing age tended to be associated with decreasing levels of CFL, but similar to contributions of sex (LMM: F_1,14_ = 1.917, *p* = 0.188) and interactions between variables, its contribution was non-significant (LMM: F_1,14_ = 3.224, *p* = 0.094). This left FDB as the only significant parameter explaining the difference with respect to CFL (LMM: F_1,16_ = 7.054, *p* = 0.017), with healthy birds consuming 22 ± 8% more of their daily food intake via CFL than feather damaging individuals (39.3 ± 19.8% versus 21.1 ± 19.6%).

**Figure 6:**
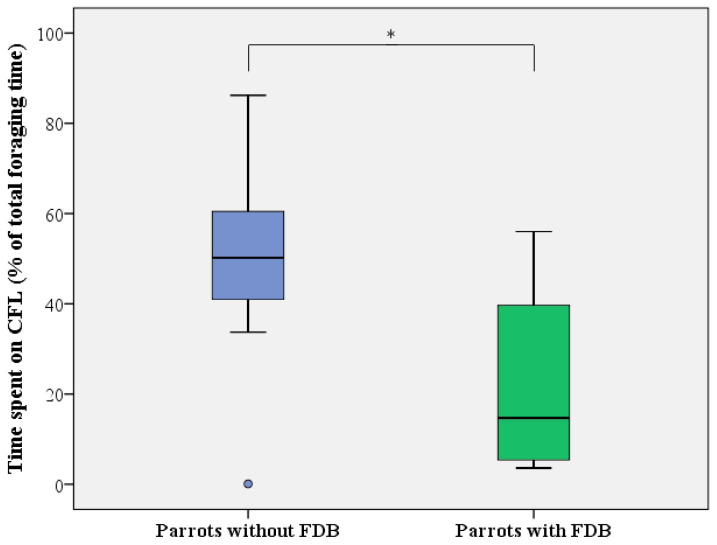
Level of CFL expressed as the time spent on foraging from the pipe feeders (in % of total foraging time) by healthy (blue) and feather damaging (green) Grey parrots. Boxes, whiskers, and dots represent the interquartile range (IQR); minimum and maximum values (1.5 × IQR); and outliers, respectively. Median values are represented by the horizontal lines within the boxes. * *p* < 0.05.

For CFL activity (i.e., time spent on manipulating and foraging from the pipe feeders relative to total foraging time), similar results were obtained, with a full mixed-effect model showing that only the presence of FDB significantly influenced investment in CFL (LMM: F_1,16_ = 10.529, *p* = 0.005), with no effects of day (LMM: F_1,31_ = 1.050, *p* = 0.362), sex (LMM: F_1,14_ = 1.904, *p* = 0.189), age (LMM: F_1,14_ = 2.550, *p* = 0.133), or any of the interactions. Based on the findings of the LMM, healthy, non-FDB birds displayed 32 ± 10% more CFL activity than feather damaging individuals (50.0 ± 22.9 versus 21.4 ± 20.0%; Figure 6).

### 3.5. Time Spent on Preening

Preening activity occurred throughout the day, with healthy, non-FDB birds spending, on average, 69 ± 42 min/day (range 24–145 min/day, *n* = 11), or 10 ± 6% of the daytime, on preening their plumage. For birds with FDB, time spent on preening was more or less comparable (mean ± SD = 76 ± 39 min/day or 12 ± 6% of the daytime, range 38–142 min/day, *n* = 10). Average bout lengths for preening measured 4.8 ± 1.7 min for birds without FDB and 3.8 ± 1.1 min for birds with FDB. No significant differences were present between the groups for preening times (Mann–Whitney U test: U = 60 *n* = 21, *p* = 0.725) or bout lengths (Mann–Whitney U test: U = 33 *n* = 21, *p* = 0.121).

### 3.6. Plumage Condition, FDB, and Its Relationship to CFL

During the acclimatization phase, no changes in feather condition were observed in any of the parrots. Similarly, no changes were observed in either the acclimatization or test period in the healthy parrots. In the feather damaging parrots, changes in plumage score were observed. Though generally small, improvements in plumage condition seemingly were higher for several of the parrots that displayed relatively higher levels of CFL (Table 2). A Pearson correlation coefficient indeed showed moderate linear correlation between change in plumage condition and time spent on CFL (r = 0.56, *n* = 10). This correlation was, however, not significant (*p* = 0.091). Post hoc power analysis on our dataset suggested that statistical significance could have been achieved for this correlation with a sample size of 14 birds.

## 4. Discussion

Both healthy and feather damaging parrots used the provided foraging devices to obtain part of their daily food intake, demonstrating that CFL occurs in Grey parrots. Similar to other studies [11,12,20], we used both the proportion of food consumed and the proportion of time spent on foraging from the pipe feeders to determine the investment in CFL by the parrots. A strong, almost linear correlation was present between these two variables. Since foraging from the pipe feeders takes more time and effort than consuming a similar amount of free food from the bowls, an increase in the proportion of food consumed from the contrafreeloading source resulted in a proportionally greater increase in CFL activity.

Previous reports already documented the existence of CFL in parrot species, including Grey parrots [22,23,24,25,89]. Orange-winged Amazon parrots (*Amazona amazonica*), however, only contrafreeloaded for larger-sized pellets offered in wooden cubes, but not for regular-sized pellets. In our study, Grey parrots did work for regular-sized pellets. Various factors may have influenced the difference in CFL observed in the two studies. In their “fuzzy model of CFL”, Inglis et al. suggested three main factors as determinants for the level of CFL, i.e., hunger (deprivation level), type of stimulus, and amount of effort required [1]. The last two factors may explain why parrots in the study of Rozek and Millam did not contrafreeload [22], while parrots in our study did. It may simply have been too much effort for the parrots to open the wooden cubes for regular pellets compared to the action that was needed to manipulate the hanging pipe feeder. In addition, the type of manipulation needed to obtain food from the pipe feeder (i.e., picking out the food from the holes) may have mimicked a more natural action than the opening of the lids of the wooden cubes, or resulted in a stimulus change that was of interest to the bird (i.e., swinging of the toy). Furthermore, differences in natural behaviour and dietary preferences of parrot species may provide an alternate explanation for the willingness to contrafreeload [95,96,109].

When given the choice between free food or manipulation of the pipe feeder, healthy Grey parrots displayed 40–50% CFL. These levels are comparable to those found for starlings [18] and domestic fowl [20]. Similarly, the total foraging times of the healthy individuals were comparable to those found in other studies [77,78,110] and somewhat approximated the minimal foraging times (i.e., 4 h/day) of wild conspecifics [71,72,73,74,75]. These high levels of CFL and large proportions of time spent on foraging indicate that parrots are internally motivated to manipulate devices to extract food, hence indicating that foraging should be considered a ‘behavioural need’, as in other ‘wild’ species [11,12,111]. The internal motivation to forage additionally mandates the provision of foraging opportunities for these birds as a standard housing requirement to benefit their welfare, as has been emphasized previously by many other authors [22,69,70,81,100,112].

As motivation to contrafreeload may change over time once the animals become more familiar with the foraging devices [113], we observed the parrots for approximately one month. Throughout this period, levels of CFL remained constant over time, indicating that birds remained motivated to use the foraging devices for at least a month. While other studies evaluating effects of enrichment have not observed changes in behaviour over time frames of up to 11 months [22,69], several authors have shown that CFL can decline following an increased number and/or duration of test trials [1,44,45]. Similarly, parrots may lose interest and motivation to contrafreeload if required to continuously perform the same type of activity without new sensory input. As such findings would emphasize the need for regular rotation or replacement of foraging opportunities, further studies would be required to determine if CFL with a single device would persist over a longer period of time.

Whereas CFL activity between different days overall remained constant, day-to-day fluctuations aside, CFL activity during the day showed a distinct pattern, with CFL occurring mainly in the morning hours (6:30 to 11 a.m.) as well as the late afternoon (2 to 5.30 p.m.). A similar foraging pattern has been observed in parrots in the wild, showing a peak of foraging activity in the morning and late afternoon, with minimal activity around noon [73,95,101,102,114]. In addition to this distinct pattern in daily activity, we observed a distinct preference for the higher-placed food sources. This may have been due to convenience as these items were placed closest to their resting position. The highest positions may have also provided the parrots with a secure look-out point, similar to the high trees that are used by wild parrots as a secure resting place [95,96,115,116]. We further noted that parrots mostly preferred to use the food bowls over manipulating the foraging toys in the first hour after being offered fresh food. Using Inglis’ model of CFL, this phenomenon may be explained by hunger, as deprivation levels will decrease CFL [1,67]. Once the physiological needs are satiated, CFL should arise, which is in accordance with what we observed in our parrots. 

Although our parrots were never completely deprived of food at any time throughout the study, often little or no food was left in their preferred food source despite filling them with 60 g of food. Based on observations during the acclimatization and the study from Lumeij and Hommers [70], this amount should have sufficed to fulfil the daily food intake from one food source. However, during the test period, when presented with multiple food sources, the birds unexpectedly consumed higher quantities of food (68 ± 14 g/day), thereby exceeding the amount that was provided per resource. It is not fully clear why the birds consumed more food compared to previously. As the birds’ body weight did not change during the test period, a true increase in food intake could only be explained by higher energy demands, e.g., due to increased traveling between food sources and/or manipulating the foraging devices. Alternatively, it may be that the increase in food intake was the result of increased spillage of food. Given the limited enrichment that was provided, the pipe feeders and even food bowls could have elicited play behaviour, as also observed by Smith et al. [23,24]. Indeed, during our analyses, we did observe birds pulling, pushing, and shaking the devices and food bowls, leading to spillage of food onto the ground, which was not necessarily consumed. Nevertheless, in our analyses, we corrected food intake for this spillage, which renders it less likely as an explanation for the increased food intake. However, we were unable to accurately account for additional spillage of food following crumbling of the pellets. This type of food wasting, which we observed frequently in our birds during the CFL trial, also occurred in a previous study [47] and is quite widespread among wild parrots, particularly during the non-breeding season [117]. With some species dropping up to half of the food that they pick, the behaviour is considered to be intentional and possibly occurs to force plants to put more energy into remaining fruits and/or lengthening the fruiting season [117]. This type of innate, species-typical behaviour could therefore be a logical explanation for the additional spillage and apparently higher food consumption observed in our study.

Based on previous studies [11,27,38,39], which suggested possible effects of age and sex on CFL in other species, we evaluated the effects of these factors on CFL activity in our parrots to exclude these from biasing the results. Similar to a study of Kuba et al. [118] in octopuses, we found no effect of age or sex on levels of exploration and interaction with foraging devices. The age-related effect, however, has been identified in many species, with younger animals displaying more CFL than older, adult animals [11,27], which might be attributed to the necessity for young, adolescent animals to gain knowledge on possible future food sources [27]. Since we observed a similar trend (*p* = 0.094), it may be that a study with a higher number of animals would have identified an age-related effect on CFL activity in parrots. As such, further explorative studies on the effects of age on CFL are warranted. 

The one factor that did contribute to the level of CFL displayed was FDB. As hypothesized, feather damaging parrots spent significantly less time on and consumed less food via CFL than healthy birds. This reduced CFL activity in feather damaging birds could be linked to the birds prioritizing the ‘compulsive behaviour’ (FDB or preening) over other activities such as foraging, as has been observed in humans with addictions and impulsive or obsessive disorders [119,120,121]. However, in contrast to CFL, preening, foraging, and food consumption appeared to be unaffected, although means for total foraging activity varied greatly between the healthy and feather damaging group, and almost reached significance (*p* = 0.09). Hence, while the difference in CFL cannot be attributed to extra time spent on preening, the tendency towards reduced foraging activity does point towards a reduced motivation to forage. An altered brain neurochemistry, such as that present in animals with ARBs [122,123], might be responsible for these changes in motivation. Imbalances in the levels of neurotransmitters such as dopamine or serotonin not only affect the occurrence of repetitive behaviour [124], but can also affect the reward systems in the brain and can enhance or reduce the reinforcing effects of stimuli, including substances (e.g., food, water, alcohol) and behaviours (e.g., foraging, preening, gambling) [125]. Additionally, these neurotransmitters play a role in decision making processes [126], including CFL. In his review, Garner [51] elucidates the role of so-called “executive systems” that are responsible for selecting and sequencing behaviour in the development of ARBs. As detailed in this review, damage to or dysfunction in these systems, which are located in the basal ganglia and prefrontal cortex, lead to inappropriate and persistent repetition of a behavioural response (‘recurrent’ perseveration) or to inappropriate, uncontrolled and extended maintenance of a thought, motivation, or activity (‘stuck-in-set’ perseveration), which are characteristic of ARBs. Neuropsychological tests such as the ‘two choice gambling task’ [55,56,57] or ‘IntraDimensional-ExtraDimensional set shifting task’ [58,127,128] can be used to assess which type of perseveration is present [51]. For example, Garner et al. [57] successfully used the ‘gambling task’ to demonstrate a link between stereotypic behaviour and ‘recurrent perseveration’. In future studies, similar neuropsychological tests may be performed to determine whether and what type of perseveration is present in feather damaging birds, thereby helping to elucidate the neuropathologic mechanisms underlying FDB, as well as determine whether the observed differences in CFL may indeed be associated with changes in the decision making process. 

The differences observed in CFL between healthy and feather damaging parrots alternatively could relate to differential effects of environmental features on birds’ behaviour. Past studies have indicated a link between FDB and stress induced by sudden changes in the household or living environment [92,129]. As we needed to move the parrots to a temporary enclosure for the purpose of this study, the stress associated with this change may have had a greater impact on the feather damaging birds’ behaviour than on that of the possibly more stress-resilient healthy birds. Similarly, the individual housing and limited availability of enrichment, which was necessary to accurately observe the individual birds’ behaviour and food consumption for shredding and prevent social facilitation and mimicking of behaviour, could have affected CFL, as previously shown by other authors [1,27,46,47,48,49]. As we were aware of the potential negative effects on the parrots’ behaviour and welfare [69,88,130,131], and to prevent potential bias from these factors as much as possible, we used an identical set-up and husbandry conditions for all birds and implemented an acclimatization period prior to the start of the study. In addition, we ensured that vocal contact between birds was maintained, provided a radio, newspaper, and soft wooden perches as sensory and occupational enrichment, and monitored parrots daily for signs indicative of stress such as initiation or worsening of FDB or stereotypic behaviours. While no obvious adverse effects were noted throughout the study in any of the parrots, it cannot be completely ruled out that any of the aforementioned factors could have differentially affected the healthy and feather damaging birds. Similarly, the knowledge gap regarding the birds’ history is a major limitation of our study, as differences in rearing and prior living conditions may not be excluded as factors influencing the level of CFL displayed [48,49,132,133,134,135]. 

While Grey parrots displayed less CFL and tended to forage less than healthy conspecifics, the foraging times of the feather damaging individuals were almost identical to those observed by Lumeij and Hommers [70]. However, while the parrots in Lumeij and Hommers’ study had no other choice than to use the pipe feeders, the parrots in our study were given a free choice between foraging from the food bowl or using the pipe feeders. The finding of similar foraging times in the ‘forced’ versus ‘voluntary’ condition indicates that the feather damaging birds may still have retained motivation to forage, yet chose to put less effort into manipulating the foraging devices in favour of spending more time on foraging from the food bowls and pellets they spilled on the ground. In addition, the plumage condition of the feather damaging parrots with higher levels of CFL seemingly improved the most, with a moderate correlation (Pearson correlation coefficient = 0.56) observed between these two parameters. However, this correlation did not reach significance (*p* = 0.091), possibly owing to the relative small sample size (*n* = 10). Moreover, the test period was relatively short (~4 weeks). Previous studies have demonstrated detectable plumage changes within this time frame [70], but similar to our study, the changes were small. In future, longer test periods are therefore recommended to detect more obvious changes. Nevertheless, the suggested correlation between CFL and improvement in feather condition as well as the finding that feather damaging parrots do contrafreeload and forage for a significant amount of time signify the importance of providing foraging opportunities to these birds as part of their standard housing.

## 5. Conclusions

The results of this study demonstrate that healthy and feather damaging Grey parrots will both work for food by manipulating foraging devices to extract food, indicating an innate, internal motivation to forage. In feather damaging birds, improvement in plumage condition seemed most likely to occur in the individuals that were willing to contrafreeload and spend longer amounts of time on foraging. These findings suggest that foraging is a ‘behavioural need’ for parrots and highlight the importance of the provision of foraging opportunities as part of standard housing requirements in a captive situation.

This study further revealed feather damaging parrots to display less CFL activity than healthy conspecifics, possibly owing to changes in the brain systems involved in decision making and perseveration of behaviour. Further research will, however, be necessary to identify whether this is indeed the case.

## Figures and Tables

**Table 1:** Score determination table for coverts and down feathers, derived from the feather scoring system of van Zeeland et al. [108].

		Down Feathers			
		No Down Removed	≤50% of Down Removed	>50% of Down Removed	All Down Removed
**Coverts**	All coverts intact	100	85	70	60
	Feather fraying or breakage	95	80	65	55
	<25% of coverts removed	90	75	60	50
	25–50% of coverts removed	80	65	50	40
	51–75% of coverts removed	70	55	40	30
	76–90% of coverts removed	60	45	30	20
	>90% of coverts removed	50	35	20	10

The table is used to assess the percentage of damage to the covert and down feathers for the different body parts (chest/flank, back, legs, and dorsal and ventral wing surface of both wings). When skin damage is present, 10 points need to be deducted from the score. Total body plumage score (0–100) is subsequently calculated as follows: 0.25 × [chest/flank] + 0.17 × [back] + 0.10 × [legs] + 0.28 × [dorsal wings] + 0.20 × [ventral wings]. Note that, for this study, only part of the feather scoring system was used, as the limited time period only allowed for detection of changes in covert and down feathers.

**Table 2:** Feather scores (score out of 100) of the parrots with feather damaging behaviour (*n* = 10) at the beginning and end of the one-month test period, in relation to contrafreeloading (CFL), expressed as percentage of total daily food intake from the pipe feeders.

Parrot	Before	After	Change	CFL Percentage
3	68	69	+1	28.8
5	60	61	+1	18.7
7	27	25	−2	17.7
9	73	75	+2	6.4
11	36	42	+6	14.3
13	46	47	+1	4.7
15	84	87	+3	40.9
17	69	75	+6	66.1
19	51	51	0	7.3
21	58	58	0	5.8

## Data Availability

The data presented in this study are available on request from the corresponding author.

## Supplementary Materials

The following supporting information can be downloaded at: https://www.mdpi.com/article/10.3390/ani13162635/s1, Figure S1: Overview of the plumage condition of the front (A) and back (B) of a Grey parrot, including details of the various body parts: chest (C), back (D), legs (E), dorsal (F,G) and ventral wing surface (H,I); Table S1: Feather score for covert and down feathers of the Grey parrot from the photographs.
