# Supplementary material for: Contrafreeloading Indicating the Behavioural Need to Forage in Healthy and Feather Damaging Grey Parrots
Source: Animals (Basel). 2023 Aug 15;13(16):2635. doi: 10.3390/ani13162635 (PMC10451555; doi:10.3390/ani13162635)
Supplement: Supplementary file 1 [file animals-13-02635-s001.zip › animals-2482389-supplementary.pdf]

Supplementary Materials – Example of Feather score

To evaluate the feather score of an individual parrot, photographs are taken of the various body parts (Figure S1A-I). Figures S1A and S1B provide an overview of the parrot, which can be used to oobtain an overview and perform a quick assessment of the feather score. For the study, however, we used close-up images of the various body parts (Figures S1C-I) to allow for randomization and mixing of the photographs during the assessment to ensure a fully blinded process. The feather scoring system as presented in Table 1 can be used to assess the feather score for the different body parts. The scores attributed to the various body parts as well as the total plumage score for the parrot in the photographs can be found in Table S1.

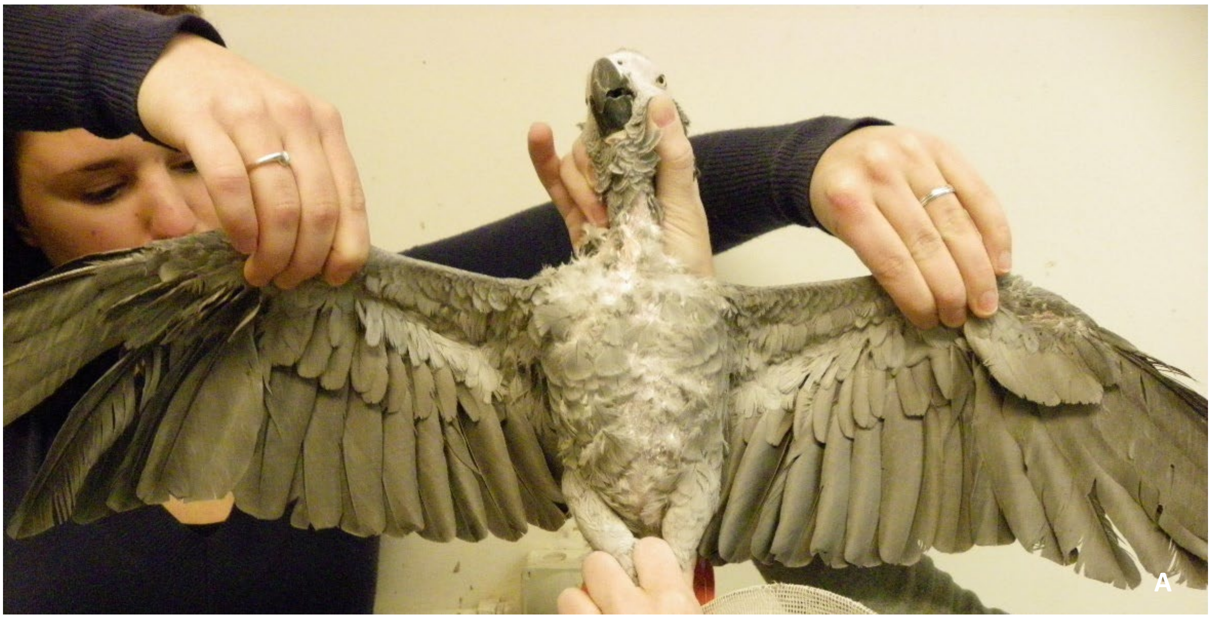

Figure S1A. Front view of a Grey parrot (*Psittacus erithacus*) with feather damaging behavior which, combined with Figure S1B can be used to obtain an overview and perform quick assessment of the feather score.

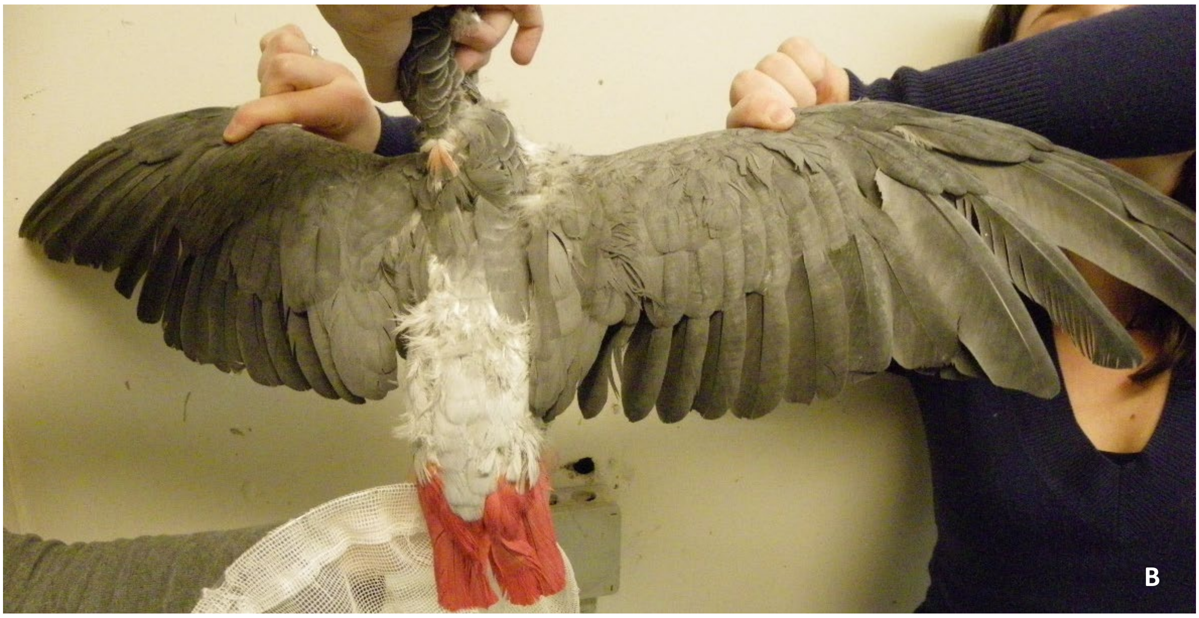

Figure S1B. Back view of a Grey parrot (*Psittacus erithacus*) with feather damaging behavior that can be used in combination with Figure S1A to obtain a full overview of the parrots plumage condition).

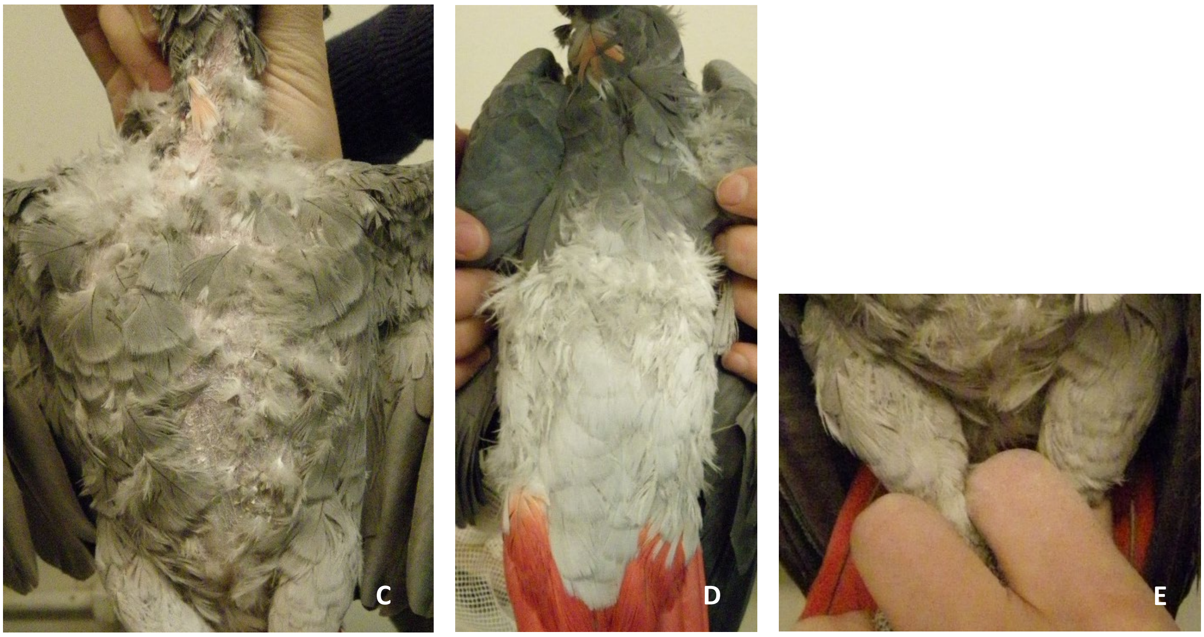

Figure S1C-E. Close-up view of the parrot's front (C) and back (D) torso, and legs (E). In this bird, between 25 and 50% of covert feathers have been removed from the front torso, as well as >50% of down feathers, resulting in a score of 50 (Table 1). On the back, <25% of the covert and no down feathers have been removed, resulting in a score of 90. On the legs, no feathers are missing, leading to a score of 100.

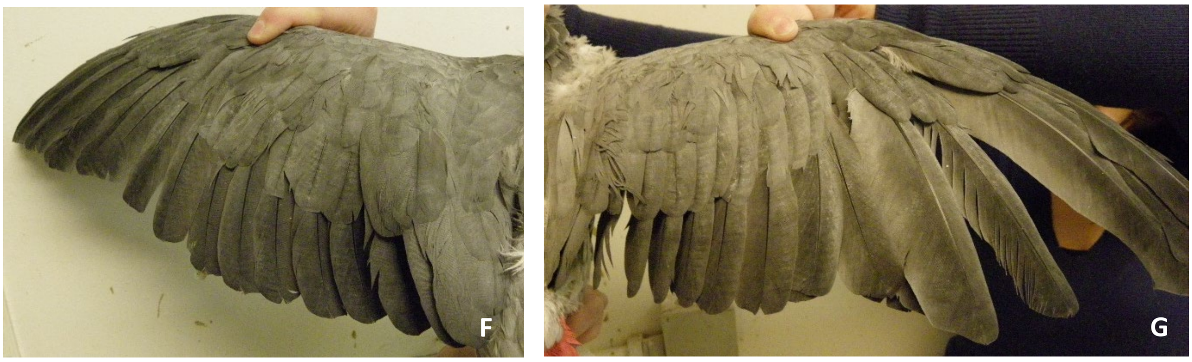

Figure S1F and G. Close-up view of the parrot's left (F) and right (G) dorsal wing surface. Similar to the legs, no damage is present to any of the covert and down feathers, leading to a score of 100.

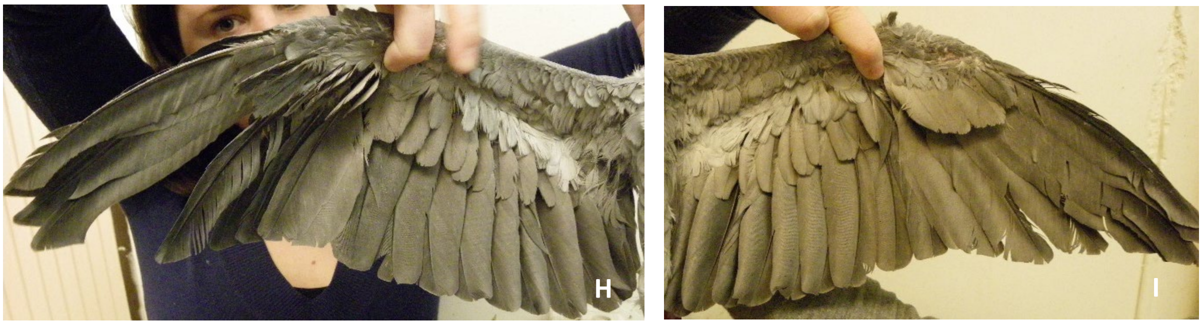

Figure S1H and I. Close-up view of the parrot's right (H) and left (I) ventral wing surface. On the outer edges of both wings, near the carpal joint, the coverts have been damaged and frayed, In addition, on both wings, local loss of down feathers, leading to exposure of bare skin can be visualized. Overall, the percentage of missing down feathers is less than 50%, leading to a score of 80.

Table S1. Feather score for covert and down feathers of the Grey parrot from the photographs. Adding up of the calculated scores results in a total plumage condition score of 81.8 for this particular parrot.

Body part	Relative contribution	Score based on Table 1A	Calculated score
Front torso	25%	50	12.5
Back torso	17%	90	15.3
Legs	10%	100	10
Dorsal wing surface	28%	100	28
Ventral wing surface	20%	80	16
**Total score**	**100%**		**81.8**
